# Assessing the Impact of Serum Ferritin on Life Skills in Children with ADHD

**DOI:** 10.3390/children12080972

**Published:** 2025-07-24

**Authors:** Merve Okuyucu, Mariam Kavakci, Merve Terzioğlu, Mehmet Enes Gökler, Mahmut Cem Tarakçıoğlu

**Affiliations:** 1 Department of Child and Adolescent Psychiatry, Ministry of Health Doğubayazıt Dr. Yaşar Eryılmaz State Hospital, Ağrı 04402, Türkiye; 2 Department of Speech and Language Therapy, Faculty of Health Sciences, Ankara Yıldırım Beyazıt University, Ankara 06760, Türkiye; mariam.kavakci@tccb.gov.tr; 3 Private Psychiatric Clinic, İstanbul 34726, Türkiye; mrv_terzioglu@yahoo.com.tr; 4 Department of Public Health, Faculty of Medicine, Ankara Yıldırım Beyazıt University, Ankara 06010, Türkiye; megokler@aybu.edu.tr; 5 Department of Child and Adolescent Psychiatry, Cerrahpasa Faculty of Medicine, Istanbul University-Cerrahpasa, İstanbul 34098, Türkiye; mc.tarakcioglu@iuc.edu.tr

**Keywords:** ferritin, ADHD, functional impairment, iron metabolism, life skills, symptom severity

## Abstract

**Background/Objectives:** This study aimed to investigate the association between serum ferritin levels and functional impairment in children with Attention Deficit Hyperactivity Disorder (ADHD). In addition, we investigated whether this relationship remained significant after controlling for core symptom severity and examined the correlations between ferritin levels and ADHD symptom levels. **Methods:** The sample included 88 children aged 6–13 years: 44 diagnosed with ADHD and 44 healthy controls (HCs) matched for age and sex. ADHD symptom severity was assessed using Turgay’s DSM-IV-Based ADHD and Disruptive Behavior Disorders Screening Scale (T-DSM-IV-S; parent-report) and the Clinical Global Impression—Severity (CGI-S) scale (clinician-rated). Functional impairment was measured using the Weiss Functional Impairment Rating Scale—Parent Report (WFIRS-P). Serum ferritin levels were determined through venous blood samples. Statistical analyses included group comparisons, Spearman correlations, and partial correlations controlling for symptom severity. **Results:** Children with ADHD had significantly lower serum ferritin levels and higher levels of both symptom severity and functional impairment compared to HCs. Ferritin levels were negatively correlated with ADHD symptom severity and with functional impairment in the Life Skills domain. However, after controlling for ADHD symptom severity, the association with Life Skills was no longer statistically significant. **Conclusions:** Ferritin levels were found to be associated with both ADHD symptom severity and functional impairment in the Life Skills domain. However, this relationship was not independent of symptom severity, suggesting that core ADHD symptoms may mediate the impact of iron status on daily functioning. Due to the study’s limitations (e.g., cross-sectional design, small sample size, gender imbalance, and lack of inflammatory and dietary data), our findings should be interpreted with caution, as they do not establish causality or resolve the ongoing inconsistencies in the literature. These results underscore the relevance of iron metabolism in the clinical presentation of ADHD and highlight the need for future research to determine whether improving iron status could serve as an adjunctive strategy in the management of functional impairments in this population.

## 1. Introduction

Attention Deficit Hyperactivity Disorder (ADHD) is among the most prevalent neurodevelopmental disorders of childhood, with global prevalence estimates ranging from 3.4% to 8% [1,2]. Defined by symptoms of inattention, hyperactivity, and impulsivity, ADHD can significantly impair academic, social, and family functioning [3]. However, these core symptoms account for only a portion of the difficulties that children with ADHD experience in their daily lives, suggesting that additional neurobiological and environmental factors contribute to the broader pattern of functional impairment [4,5].

Functional impairment in ADHD refers to disruptions in everyday activities that extend beyond the diagnostic criteria, affecting multiple domains such as academic performance, family relationships, social interactions, and daily living skills. These impairments are often more disabling than the symptoms themselves and are crucial determinants of long-term outcomes [6,7].

Among the biological systems implicated in ADHD, iron metabolism has received increasing attention due to iron’s critical role in brain development, myelination, and dopaminergic neurotransmission [8,9,10]. Ferritin, as the primary intracellular iron storage protein, regulates dopamine synthesis through its influence on tyrosine hydroxylase, the rate-limiting enzyme in dopamine production. Given the established link between dopamine dysfunction and ADHD, alterations in iron homeostasis may be associated not only with core behavioral symptoms but also with broader deficits in functional abilities, particularly in tasks requiring executive control and self-regulation, such as life skills and organization.

Evidence suggests that iron deficiency during childhood—even in the absence of anemia—can adversely affect cognitive, motor, and emotional functioning [11,12,13]. Several neurobiological mechanisms have been proposed to explain the role of iron in ADHD, including its involvement in dopamine synthesis via tyrosine hydroxylase, alterations in dopamine transporter and receptor activity in the striatum, disruptions in GABAergic transmission, and reduced brain iron content—particularly in the thalamus and basal ganglia—observed in neuroimaging studies [11,14,15]. Nevertheless, meta-analytic evidence indicates that children with ADHD have significantly lower serum ferritin levels than healthy controls (HCs) [11,16]. Most of the existing literature has examined ferritin levels in relation to ADHD symptom severity, typically using measures of hyperactivity, impulsivity, and inattention as outcome variables. However, the majority of these studies have focused on associations between ferritin and symptom severity—particularly hyperactivity and inattention—while largely overlooking the relationship between ferritin and functional outcomes. Importantly, no studies to date have systematically examined whether ferritin levels are linked to impairments in specific domains such as life skills or social functioning, independent of ADHD symptoms.

The present study aims to investigate whether serum ferritin levels are associated not only with ADHD symptoms but also with functional impairments, particularly in areas such as life skills. By statistically controlling ADHD symptom severity, we seek to determine whether ferritin contributes independently to functional difficulties. This approach moves beyond symptom-focused models of ADHD to consider the biological underpinnings of daily functioning—an area that has received limited empirical attention. To our knowledge, this is the first study to examine the link between ferritin and specific domains of functional impairment in ADHD.

Hypotheses

(1) We hypothesize that children diagnosed with ADHD will exhibit significantly lower serum ferritin levels and greater functional impairment across domains when compared to HCs. This includes both elevated ADHD symptomatology (inattention, hyperactivity, impulsivity) and increased deficits across functioning domains such as family life, academic performance, and peer interactions.
(2) We hypothesize that serum ferritin levels will remain significantly associated with both overall and specific domains of functional impairment, even after statistically controlling for ADHD symptom severity. This would suggest an independent contribution of ferritin to functional difficulties beyond its relation to core symptoms.

## 2. Materials and Methods

### 2.1. Study Design

This cross-sectional study was conducted at the Child and Adolescent Psychiatry Outpatient Clinic of the Ministry of Health Doğubayazıt Dr. Yaşar Eryılmaz State Hospital between October 2024 and January 2025. The study targeted school-aged children (6–13 years).

A priori power analysis was performed using G*Power version 3.1.9.7 [17]. Based on an expected medium effect size (Cohen’s d = 0.62), derived from means and standard deviations reported in a previous study [18], with α = 0.05 and power = 0.90, the required sample size was calculated to be 90 participants (45 per group). Although this was the initial target, the final sample consisted of 88 children due to participant availability constraints.

Ethical approval for the study was obtained from the Ethics Committee of Ağrı İbrahim Çeçen University (Date: 27 September 2024; Approval Code: E.112433). All procedures were conducted in accordance with the Declaration of Helsinki (revised 2000). Verbal assent was obtained from child participants, and written informed consent was obtained from their legal guardians.

Initially, 72 children with ADHD and 68 HCs were invited to participate. Children in the ADHD group met the diagnostic criteria for ADHD according to the Diagnostic and Statistical Manual of Mental Disorders, Fifth Edition (DSM-5) [3]. The HC group was recruited from children attending family medicine clinics for routine health check-ups and was confirmed to be free of psychiatric disorders via standardized psychiatric evaluations.

Inclusion criteria for all participants were as follows:

1. Aged between 6 and 13 years;
2. Provision of informed consent.

Exclusion criteria for both groups were as follows:

1. Any diagnosed chronic medical condition (e.g., metabolic, genetic, endocrinologic, neuromuscular, neurological, or inflammatory disorders), confirmed by clinical records and parent interviews;
2. Acute or chronic systemic illnesses (e.g., autoimmune diseases, chronic infections);
3. Suspected intellectual disability based on developmental and academic history;
4. Infection within the past month;
5. Use of iron supplements within the past month;
6. Hemoglobin (Hb) levels below 12 g/dL;
7. Use of any medication or supplements within the last month (except ADHD treatment in the ADHD group).

Additional criteria for the ADHD group included the following:

1. A confirmed diagnosis of ADHD;
2. Absence of any comorbid psychiatric diagnosis, except for Specific Learning Disorder (SLD) or Oppositional Defiant Disorder (ODD).

For the HC group, the inclusion criteria were as follows:

1. Absence of current or past psychiatric disorders;
2. No history of psychiatric treatment.

At the time of assessment, eight participants in the ADHD group were undergoing treatment with methylphenidate, whereas the remaining participants were drug-naive.

The Schedule for Affective Disorders and Schizophrenia for School-Age Children—Present and Lifetime Version (K-SADS-PL-DSM-5) was administered to all participants to assess for psychiatric disorders [19]. Diagnoses were based on structured clinical interviews using DSM-5 criteria [3]. In cases of suspected SLD, academic performance and visual-spatial assessments were used to confirm the diagnosis. All evaluations were performed by a certified child and adolescent psychiatrist. The diagnostic process was supervised by an academic advisor (associate professor in child and adolescent psychiatry), who was consulted throughout case selection.

**Figure 1:**
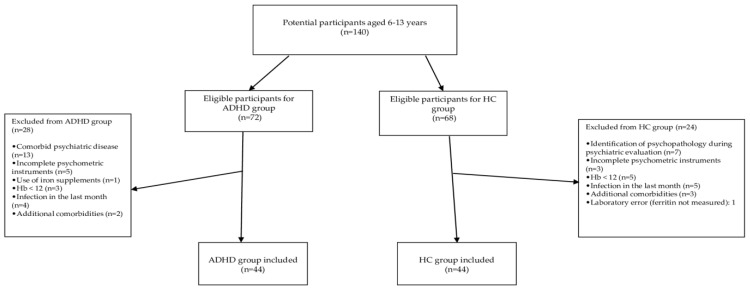
Flowchart of participant inclusion and exclusion criteria.

Ultimately, 44 children with ADHD and 44 age-matched HCs were included in the study. The participant flowchart is presented in Figure 1.

Participants first completed a structured questionnaire providing sociodemographic and clinical information. A licensed child and adolescent psychiatrist conducted psychiatric evaluations for all participants using K-SADS-PL-DSM-5 [19]. This assessment confirmed ADHD diagnoses in the clinical group and ensured the absence of psychiatric disorders in the HC group.

Symptom severity was evaluated using the Clinical Global Impression—Severity Scale (CGI-S), administered by the clinician [20]. Functional impairment was assessed using the Weiss Functional Impairment Rating Scale—Parent Report (WFIRS-P), adapted and validated for Turkish populations by Tarakçıoğlu et al. (2015) [21]. ADHD symptom severity was measured using Turgay’s DSM-IV-Based ADHD and Disruptive Behavior Disorders Screening Scale (T-DSM-IV-S), originally developed by Turgay (1995; DSM-IV: Diagnostic and Statistical Manual of Mental Disorders, Fourth Edition) [22]. All parent-report questionnaires were completed independently; however, when families reported difficulties with reading or comprehension, assistance was provided by the clinician.

Following psychometric assessments, participants’ height and weight were measured by a certified dietitian. Venous blood samples were collected from the antecubital vein using yellow-capped gel tubes. Hemogram measurements were performed promptly after collection on EDTA-treated blood samples using fully automated blood count devices (Mindray BC 6800), employing electrical impedance and optical light scattering techniques. Serum ferritin levels were analyzed using the immunospectrometric method on the Beckman Coulter UniCel DxI 800 Immunoassay System. The Access Ferritin Assay is a chemiluminescent, two-site sandwich immunoenzymatic assay, in which ferritin molecules bind simultaneously to paramagnetic particle-bound monoclonal anti-ferritin antibodies and enzyme-labeled polyclonal anti-ferritin antibodies. The resulting chemiluminescent signal was measured and compared against a calibration curve. The assay had a detection range of 0.2–1500 ng/mL (up to 15,000 ng/mL with onboard dilution), with a total coefficient of variation below 10% across clinical ranges. All measurements were performed in the hospital’s central biochemistry laboratory under standard quality control procedures.

### 2.2. Assessment Tools

#### 2.2.1. Sociodemographic Data Form

This form was developed by the research team to collect information on the sociodemographic characteristics of the child, family, and primary caregivers. It also included questions regarding early developmental risk factors.

#### 2.2.2. Schedule for Affective Disorders and Schizophrenia for School-Age Children—Present and Lifetime Version (K-SADS-PL-DSM-5)

The K-SADS-PL-DSM-5 is a semi-structured diagnostic interview designed to assess psychiatric disorders in children aged 6 to 18 years, based on DSM-5 criteria [19]. It is administered by certified child and adolescent psychiatrists who integrate information from both the child and caregiver to evaluate the presence and severity of psychiatric symptoms. The Turkish version of the scale has been validated [23].

#### 2.2.3. Weiss Functional Impairment Rating Scale—Parent Report (WFIRS-P)

The WFIRS-P was developed by Weiss et al. (2007) to assess functional impairment in six domains: Family, School and Learning, Life Skills, Self-Concept, Social Activities, and Risky Activities [24]. It contains 50 items rated on a 4-point Likert scale (0 = never or not at all to 3 = very often or very much). Mean scores are calculated for each subdomain, with higher scores indicating greater impairment. The Turkish adaptation has demonstrated excellent reliability, with Cronbach’s alpha = 0.93 and test–retest correlation = 0.93 [21]. In this study, the scale was completed by parents.

#### 2.2.4. Clinical Global Impression—Severity Scale (CGI-S)

The CGI-S is a clinician-rated tool that assesses overall illness severity on a 7-point scale ranging from 1 (not at all ill) to 7 (among the most extremely ill) [20]. Only the severity subscale was used in this study to evaluate the global clinical impression of each participant.

#### 2.2.5. Turgay’s DSM-IV-Based Disruptive Behavior Disorders Screening and Rating Scale (T-DSM-IV-S)

This parent- and teacher-rated scale was developed by Turgay (1995) and consists of 41 items based on DSM-IV criteria, including: inattention (9 items), hyperactivity (6), impulsivity (3), Oppositional Defiant Disorder (8), and conduct disorder (15) [22]. Each item is rated on a 4-point Likert scale from 0 (not at all) to 3 (very much). The Turkish version was validated by Ercan et al. (2001) [25]. In this study, the primary caregivers completed the T-DSM-IV-S to assess ADHD symptom severity.

### 2.3. Statistical Analyses

All statistical analyses were conducted using IBM SPSS Statistics version 26. The normality of distribution for continuous variables was evaluated using the Shapiro–Wilk test. As most variables violated the assumption of normality, non-parametric statistical methods were employed throughout the analysis.

Group comparisons between the ADHD and HC groups were conducted using the Mann–Whitney U test for continuous variables and the chi-square (χ^2^) test for categorical variables. To aid interpretation of the group differences, effect sizes were calculated using rank biserial correlation coefficients and reported alongside *p*-values. Effect sizes were interpreted according to standard conventions, with values around 0.1 indicating a small effect, 0.3 a medium effect, and 0.5 or greater a large effect.

To examine associations between serum ferritin levels, ADHD symptom severity (as measured by the T-DSM-IV-S and CGI-S), and functional impairment (as measured by the WFIRS-P), Spearman’s rank-order correlation coefficients were calculated. For each correlation, the proportion of explained variance (r^2^) was also computed by squaring the Spearman correlation coefficient. These values were reported to provide a clearer understanding of how much variance in symptoms or functioning could be accounted for. In addition, partial correlation analyses were performed to assess the relationship between serum ferritin levels and functional impairment while controlling for ADHD symptom severity. Explained variance (r^2^) values were also reported for these partial correlations.

All statistical tests were two-tailed, and the significance level was set at *p* < 0.05.

## 3. Results

### 3.1. Demographic and Clinical Profiles of ADHD and HC Groups

A total of 88 children were included in the final analysis, with 44 diagnosed with ADHD and 44 HCs. The groups were compared on sociodemographic and clinical variables (see Table 1).

Gender distribution differed significantly between the groups: 81.8% of the ADHD group were boys, while 63.6% of the HC group were girls (χ^2^(1) = 18.803, *p* < 0.001). However, the groups were comparable in age, height, and weight (all *p* > 0.05). Similarly, there were no significant differences in grade level (χ^2^(7) = 8.466, *p* = 0.389) or household income (χ^2^(2) = 2.911, *p* = 0.233).

As expected, psychiatric comorbidities were present only in the ADHD group (34.1% vs. 0%, χ^2^(1) = 18.082, *p* < 0.001). Specifically, 9.1% had ODD and 25.0% had SLD. No significant differences were found between groups in the history of premature birth (*p* = 0.315), and none of the participants had chronic illness.

Table 1 presents detailed comparisons across demographic and clinical variables.

### 3.2. Functional Impairment, Symptom Severity, and Ferritin Levels

Children with ADHD demonstrated significantly greater functional impairment across all WFIRS-P domains and in the total score compared to HCs (all *p* < 0.001). They also had higher symptom severity, as indicated by both parent-reported (T-DSM-IV-S) and clinician-rated (CGI-S) assessments (*p* < 0.001 for both).

Biochemically, serum ferritin levels were significantly lower in the ADHD group than in HCs (median = 21.00 vs. 28.00 ng/mL; *p* = 0.020), while Hb levels did not differ significantly between groups (*p* = 0.512).

Table 2 provides detailed comparisons of functional impairment scores, symptom severity, and biochemical parameters between the groups.

### 3.3. Correlation Between Functional Impairment, Symptom Severity, and Ferritin Levels

In the ADHD group, functional impairment was strongly and positively correlated with symptom severity. The WFIRS-P total score was highly correlated with both the T-DSM-IV-S total score (r = 0.924, *p* < 0.001) and the CGI-S score (r = 0.856, *p* < 0.001). Furthermore, the T-DSM-IV-S and CGI-S scores were also strongly correlated with each other (r = 0.862, *p* < 0.001), indicating consistency across clinical symptom severity assessments.

Serum ferritin levels demonstrated significant negative correlations with all three primary clinical measures: functional impairment (WFIRS-P total score, r = −0.280, *p* = 0.008), parent-reported symptom severity (T-DSM-IV-S, r = −0.289, *p* = 0.006), and clinician-rated global severity (CGI-S, r = −0.288, *p* = 0.006).

Hb levels showed no significant associations with clinical scale scores, although they were positively correlated with ferritin levels (r = 0.357, *p* = 0.001).

The results are presented in Table 3.

### 3.4. Correlations Within the ADHD Group

Within the ADHD group (*n* = 44), greater symptom severity was significantly associated with increased functional impairment. The WFIRS-P total score correlated strongly with the T-DSM-IV-S total score (r = 0.764, *p* < 0.001) and moderately with the CGI-S score (r = 0.487, *p* = 0.001). A significant correlation was also found between the T-DSM-IV-S and CGI-S scores (r = 0.453, *p* = 0.002), indicating consistency between symptom severity measures.

Serum ferritin levels were negatively correlated with the T-DSM-IV-S total score (r = −0.341, *p* = 0.024), but showed no significant associations with the WFIRS-P total score (r = −0.156, *p* = 0.312) or the CGI-S score (r = −0.206, *p* = 0.180).

Hb levels were not significantly associated with any clinical scores, but were positively correlated with ferritin levels (r = 0.378, *p* = 0.011).

Full correlation coefficients are reported in Table 4.

### 3.5. Associations Between Ferritin Levels and Functional Impairment Domains

In the ADHD group, serum ferritin levels showed a significant negative correlation specifically with the WFIRS-P Life Skills domain (r = −0.382, *p* = 0.010). However, correlations with other functional domains—Family, School, Self-Concept, Social Activities, and Risky Activities—were not statistically significant (all *p* > 0.05).

Full results of the associations between ferritin levels and the functional impairment domains are reported in Table 5.

### 3.6. Partial Correlations Between Ferritin Levels and Functional Impairment, Controlling for Symptom Severity

To evaluate whether serum ferritin levels were associated with functional impairment independent of ADHD symptom severity, partial correlation analyses were conducted while controlling for the T-DSM-IV-S total score.

After adjustment for symptom severity, ferritin levels were not significantly correlated with the WFIRS-P total score or any of its subdomains (all *p* > 0.05).

These findings suggest that the previously observed associations between ferritin and functioning—particularly in the Life Skills domain—may be largely explained by the severity of ADHD symptoms rather than representing an independent contribution of ferritin to functional outcomes.

Detailed partial correlation coefficients are presented in Table 6.

## 4. Discussion

This study examined the associations between serum ferritin levels, ADHD symptom severity, and functional impairment in a sample of children diagnosed with ADHD compared to HCs. The results revealed that lower ferritin levels were significantly associated with higher symptom severity and greater functional impairment across multiple domains, particularly in life skills. However, when ADHD symptom severity was statistically controlled, these associations were no longer significant, suggesting that the observed relationships may be mediated by core ADHD symptoms. These findings extend previous literature on the biological underpinnings of ADHD by highlighting the role of iron metabolism in clinical and functional outcomes, while also emphasizing the importance of symptom severity as a potential confounding factor. A key finding of this study was the significant association between lower serum ferritin levels and greater functional impairment in children with ADHD, particularly in the Life Skills domain. Importantly, all participants had Hb levels ≥12 g/dL, indicating that this relationship is unlikely to reflect anemia and may instead represent a marker of subclinical iron deficiency.

Previous research has linked low ferritin levels to cognitive deficits [26,27], attentional difficulties [26], and sleep disturbances [12], all of which may impact functioning. However, in the present study, when ADHD symptom severity was statistically controlled, the relationship between ferritin and functional impairment was no longer significant. This suggests that ferritin’s association with functioning may be mediated by symptom severity, rather than representing an independent contribution to functional outcomes.

Although this limits the interpretation of ferritin as a standalone biomarker of impairment, it supports its potential role as a contributing factor within broader biological pathways underlying ADHD. Further research is warranted to explore whether ferritin levels may indirectly influence daily functioning via effects on core ADHD symptoms.

Another notable finding of this study was the significant association between serum ferritin levels and the Life Skills subdomain of functional impairment in children with ADHD. Life Skills refer to competencies such as personal hygiene, time management, homework completion, and daily routines—domains that are critical for independent functioning. While the association between ferritin and Life Skills impairment was initially significant, it did not remain so after statistically controlling for ADHD symptom severity. This suggests that the impact of ferritin on life skills may be mediated or confounded by the severity of core ADHD symptoms.

This finding aligns with prior research showing a dissociation between symptom reduction and functional recovery in ADHD. For example, Coghill et al. (2019) reported that symptom improvements did not necessarily translate into functional gains, while Weiss et al. (2018) similarly observed that stimulant medication often reduced symptoms without fully resolving functional difficulties [4,28]. Additionally, Craig et al. (2020) found that sleep disturbances—an outcome linked to low iron stores—were associated with poor life skills functioning regardless of ADHD symptom severity [29]. Collectively, these studies underscore the importance of considering biological and environmental contributors to everyday functioning in ADHD, beyond symptom control alone.

From a neurobiological perspective, ferritin regulates iron storage, which is essential for dopamine synthesis via tyrosine hydroxylase [8,10]. Dopaminergic dysregulation is a central mechanism in ADHD pathophysiology [30], and iron deficiency may impair dopamine production, affecting prefrontal and striatal circuits and areas involved in executive functions and behavioral regulation [9,31]. Therefore, although our results do not confirm a direct effect of ferritin on functional outcomes when accounting for symptom severity, the Life Skills domain may be particularly susceptible to subtle neurobiological influences that warrant further investigation.

Supporting this possibility, domains such as Life Skills and Self-Concept are known to improve more slowly and inconsistently than academic or behavioral functioning [28,32]. It is plausible that biological factors like iron regulation may exert a delayed or indirect influence on these areas, which are not easily targeted by conventional ADHD treatments. Future longitudinal studies should examine whether improving ferritin levels through dietary or pharmacologic means may positively affect functional outcomes, particularly in areas requiring self-regulation and executive control.

Consistent with prior meta-analyses [11,16], our results showed significantly lower ferritin levels in children with ADHD compared to HCs. However, the literature remains mixed: some studies report no differences [33,34] or even elevated ferritin in ADHD [35]. These discrepancies may be attributed to variations in sample characteristics, diagnostic tools, medication status, or exclusion criteria. For instance, the study by Lukovac et al. (2024) included only boys and did not exclude participants with recent infections or supplement use, factors that could affect ferritin levels [35]. In contrast, our study applied strict exclusion criteria, including removal of participants with recent infections, iron supplementation, or low Hb levels, thereby increasing confidence in the biological specificity of our ferritin findings.

To contextualize our findings, a summary of major studies examining ferritin levels in pediatric ADHD populations is provided in Supplementary Table S1.

Moreover, we observed moderate negative correlations between ferritin levels and both parent-reported (T-DSM-IV-S) and clinician-rated (CGI-S) ADHD symptom severity. While some prior studies have reported similar findings [36,37], others have found only weak or non-significant associations [34,38]. One possible explanation for these inconsistencies is methodological: many studies categorized ferritin values using arbitrary cutoffs, whereas our use of continuous ferritin scores may have captured more nuanced variation in iron-related biology.

An additional consideration is the potential effect of stimulant medication on ferritin levels. Within our ADHD sample, eight children were receiving methylphenidate treatment, while the rest were drug-naive. Although our study did not directly assess treatment effects, a recent randomized controlled trial by Rosenau et al. (2022) reported decreased ferritin levels following methylphenidate withdrawal [39]. The authors proposed dopaminergic regulation and changes in transporter density as possible mediators. These findings suggest that stimulant exposure may influence systemic iron markers, warranting further investigation.

Emerging evidence suggests that early-life iron deficiency may affect brain development, especially in dopaminergic and myelination-related regions such as the prefrontal cortex and basal ganglia [9,40]. These developmental alterations could have long-term consequences on cognitive and behavioral outcomes. Although our study did not assess early-life ferritin levels, it is plausible that suboptimal iron during critical periods of neurodevelopment may contribute to the functional difficulties observed in ADHD. Understanding the potential role of early iron deficiency in the development of ADHD symptoms and related impairments is an important area for future research.

Finally, it should be noted that the ADHD group in our sample was predominantly male. Given known sex differences in iron metabolism and ADHD presentation [41,42,43], this may limit the generalizability of our findings. Future studies should stratify analyses by sex to explore whether the relationship between ferritin and ADHD-related outcomes differs across males and females.

In summary, this study contributes to the growing body of research on ferritin’s role in ADHD by identifying significant associations between serum ferritin levels, symptom severity, and functional impairment, particularly in life skills functioning. While these relationships did not remain significant after controlling for symptom severity, the findings suggest that ferritin may be involved indirectly in the clinical presentation of ADHD, possibly through its influence on neurobiological systems that underlie executive and self-regulatory functions.

Several limitations of this study should be acknowledged. First, the cross-sectional design precludes causal inferences and does not allow for determining the directionality of the observed associations between serum ferritin levels, ADHD symptom severity, and functional outcomes. Second, ferritin was assessed at a single time point, and potential fluctuations over time or across seasons were not evaluated.

Third, the study was conducted at a single clinical center with a relatively homogeneous socioeconomic sample, which may limit the generalizability of the findings. Fourth, although most blood samples were collected in the morning and following fasting, the timing of sample collection was not strictly standardized, and fasting status was not systematically controlled.

Fifth, the ADHD group was predominantly male, limiting the applicability of results across sexes, especially considering known sex differences in both iron metabolism and ADHD symptom expression. Additionally, pubertal status—which is known to affect ferritin levels—was neither measured nor statistically accounted for.

While symptom severity was assessed using both clinician-rated (CGI-S) and parent-reported (T-DSM-IV-S) measures, functional impairment was evaluated exclusively through parent-report (WFIRS-P). This reliance on a single informant for assessing functional outcomes may introduce reporting bias, as parental perceptions can be shaped by contextual or subjective influences.

Although all children drank municipally supplied tap water, we did not measure environmental metal exposures, which could confound ferritin–ADHD associations.

Within the ADHD group, Life Skills scores were uniformly high, precluding stratification into high- versus low-functioning subgroups; future research with larger and more diverse samples may address this. We encourage future studies to adopt more refined groupings.

Sleep habits and dietary/nutritional status were not measured; these factors may confound ferritin–ADHD associations and should be included in future studies. Although participants with chronic medical conditions were excluded, subclinical inflammation was not assessed using objective biomarkers like CRP or IL-6. While white blood cell counts were reviewed and found to be within normal limits in nearly all participants, we cannot entirely rule out residual confounding from low-grade inflammation.

Lastly, while serum ferritin is widely used as a peripheral marker of iron status, it may not reliably reflect brain iron content, particularly in dopaminergic regions relevant to ADHD. Therefore, caution is warranted when interpreting neurobiological implications from serum ferritin levels alone.

To strengthen the robustness and generalizability of findings, future studies should employ longitudinal designs, ensure more balanced samples, control for biological variables such as puberty and fasting, and incorporate multi-informant assessments. Longitudinal or interventional research will be particularly valuable in clarifying whether low iron status contributes to functional difficulties or whether such difficulties impact iron metabolism through behavioral or dietary pathways.

Given the accessibility and clinical relevance of serum ferritin measurements, future studies should investigate its utility as a complementary biomarker in ADHD assessment, particularly in identifying subgroups of children who may be at greater risk for functional challenges. It is also crucial to determine whether targeting iron status can produce sustained improvements in both symptomatology and daily functioning.

## 5. Conclusions

In conclusion, this study contributes to the emerging literature on the role of iron metabolism in ADHD by demonstrating significant associations between serum ferritin levels, symptom severity, and functional impairment, particularly within the domain of life skills. Although these associations were no longer significant after controlling for symptom severity, the findings suggest that ferritin may influence ADHD-related functioning indirectly, possibly through its impact on core symptomatology.

The observed pattern highlights the importance of considering biological contributors such as iron status in the broader clinical picture of ADHD. While the results do not support ferritin as a standalone biomarker of functional outcomes, they raise the possibility that low ferritin levels may exacerbate self-regulatory challenges. Future longitudinal and interventional studies are needed to clarify the causal pathways involved and to determine whether correcting iron deficiency can improve both symptom severity and everyday functioning in children with ADHD.

## Figures and Tables

**Table 1:** Comparison of demographic and clinical characteristics between ADHD and HC groups (*n* = 88).

Variables	Categories	*n*/% or Median (P25–P75)	*n*/% or Median (P25–P75)	X^2^/Z	*p*
		ADHD (*n* = 44)	HC (*n* = 44)		
Gender	Girl	8 (18.2)	28 (63.6)	18.803	<0.001
	Boy	36 (81.8)	16 (36.4)		
Age		9.00 (7.05–10.09)	8.56 (8.02–10.04)	−0.555	0.579
Height (cm)		136.00 (127.00–144.00)	133.50 (125.50–142.50)	−0.639	0.523
Weight (kg)		28.50 (24.63–37.67)	27.45 (23.25–34.70)	−1.094	0.274
Grade Level	Kingergarten	0 (0.0)	1 (2.3)	8.466	0.389
	1st grade	6 (13.6)	3 (6.8)		
	2nd grade	9 (20.5)	4 (9.1)		
	3rd grade	7 (15.9)	14 (31.8)		
	4th grade	7 (15.9)	7 (15.9)		
	5th grade	8 (18.2)	7(15.9)		
	6th grade	4 (9.1)	3 (6.8)		
	7th grade	3 (6.8)	3 (6.8)		
	8th grade	0 (0.0)	2 (4.5)		
Monthly Income	Below minimum wage	20 (45.5)	21 (47.7)	2.911	0.233
	Minimum wage-100 thousand TL	19 (43.2)	22 (50.0)		
	More than 100 thousand TL	5 (11.4)	1 (2.3)		
Additional Psychiatric Disorder	None	29 (65.9)	44 (100.0)	18.082	<0.001
	ODD	4 (9.1)	0 (0.0)		
	SLD	11 (25.0)	0 (0.0)		
Premature Birth	None	43 (97.7)	44 (100.0)	1.011	0.315
	Present	1 (2.3)	0 (0.0)		
Chronic Illness	None	44 (100.0)	44 (100.0)	-	-
	Present	0 (0.0)	0 (0.0)		

ADHD: Attention Deficit Hyperactivity Disorder; HC: healthy controls; cm: centimeters; kg: kilograms; ODD: Oppositional Defiant Disorder; SLD: Specific Learning Disorders; TL: Turkish Liras. Values are presented as *n* (%) for categorical variables and as median (interquartile range, P25–P75) for continuous variables. Group differences were assessed using chi-square tests (X^2^) for categorical variables and Mann–Whitney U tests (Z) for continuous variables.

**Table 2:** Comparison of scale scores and biochemical parameters between the ADHD group and the HC group (*n* = 88).

Variables	Median (P25–P75)	Median (P25–P75)	Z	*p*	Effect Size
	ADHD (*n* = 44)	HC (*n* = 44)			
WFIRS-P Family	9.50 (4.50–16.00)	1.00 (0.00–2.00)	−6.99	<0.001	0.8585
WFIRS-P School	12.00 (8.00–14.50)	0.00 (0.00–1.00)	−7.925	<0.001	0.9582
WFIRS-P Life Skills	10.00 (5.50–15.00)	2.00 (0.00–5.00)	−6.075	<0.001	0.7490
WFIRS-P Self-Concept	1.00 (0.00–3.00)	0.00 (0.00–0.00)	−4.457	<0.001	0.4602
WFIRS-P Social Activities	6.50 (4.00–10.00)	0.00 (0.00–1.00)	−7.271	<0.001	0.8750
WFIRS-P Risky Activities	3.00 (1.50–3.00)	0.00 (0.00–0.00)	−6.816	<0.001	0.7991
WFIRS-P Total	44.00 (29.50–59.00)	4.00 (1.00–9.00)	−7.904	<0.001	0.9778
T-DSM-IV-S Total	48.50 (35.50–60.50)	2.00 (1.00–5.50)	−8.006	<0.001	0.9892
CGI-S	5.00 (4.00–5.00)	1.00 (1.00–1.00)	−8.689	<0.001	1.0000
Ferritin	21.00 (13.50–30.00)	28.00 (22.00–40.00)	−2.334	0.020	0.2887
Hb	14.15 (13.40–14.50)	14.05 (13.60–14.70)	−0.656	0.512	0.0811
WBC	8.24 (7.37–10.07)	8.81 (6.83–9.58)	1.025	0.637	0.0589

ADHD: Attention Deficit Hyperactivity Disorder; HC: healthy controls; CGI-S: Clinical Global Impression Scale—Severity; Hb: Hemoglobin; T-DSM-IV-S: Turgay’s DSM-IV Based Attention Deficit Hyperactivity Disorder and Disruptive Behavior Disorder Screening Scale; WBC: white blood cell; WFIRS-P: Weiss Functional Impairment Rating Scale—Parent Form. Values are expressed as medians with interquartile ranges (P25–P75). Z values represent the results of the Mann–Whitney U test. Group differences were considered statistically significant at *p* < 0.05. WBC was included in the analysis to help rule out potential underlying infections or inflammatory conditions that could influence ferritin levels.

**Table 3:** Correlations between clinical scale scores and ferritin levels in children with ADHD (*n* = 44).

		WFIRS-P Total	T-DSM-IV-S Total	CGI-S	Ferritin	Hb
WFIRS-P Total	R	1.000	0.924 **	0.856 **	−0.280 **	−0.053
	r^2^	–	0.854	0.733	0.078	0.003
T-DSM-IV-S Total	R	0.924 **	1.000	0.862 **	−0.289 **	−0.131
	r^2^	0.854	–	0.743	0.084	0.017
CGI-S	R	0.856 **	0.862 **	1.000	−0.288 **	−0.025
	r^2^	0.733	0.743	–	0.083	0.001
Ferritin	R	−0.280 **	−0.289 **	−0.288 **	1.000	0.357 **
	r^2^	0.078	0.084	0.083	–	0.127
Hb	R	−0.053	−0.131	−0.025	0.357 **	1.000
	r^2^	0.003	0.017	0.001	0.127	–

** *p* < 0.01. ADHD: Attention Deficit Hyperactivity Disorder; CGI-S: Clinical Global Impression Scale—Severity; Hb: Hemoglobin; T-DSM-IV-S: Turgay’s DSM-IV Based Attention Deficit Hyperactivity Disorder and Disruptive Behavior Disorder Screening Scale; WFIRS-P: Weiss Functional Impairment Rating Scale—Parent Form. r^2^ = explained variance.

**Table 4:** Correlations between functional impairment and clinical symptoms in the ADHD group (*n* = 44).

		WFIRS-P Total	T-DSM-IV-S Total	CGI-S	Ferritin	Hb
WFIRS-P Total	R	1.000	0.764 **	0.487 **	−0.156	0.142
	r^2^	–	0.584	0.237	0.024	0.020
T-DSM-IV-S Total	R	0.764 **	1.000	0.453 **	−0.341 *	−0.137
	r^2^	0.584	–	0.205	0.116	0.019
CGI-S	R	0.487 **	0.453 **	1.000	−0.206	0.176
	r^2^	0.237	0.205	–	0.042	0.031
Ferritin	R	−0.156	−0.341 *	−0.206	1.000	0.378 *
	r^2^	0.024	0.116	0.042	–	0.143
Hb	R	0.142	−0.137	0.176	0.378 *	1.000
	r^2^	0.020	0.019	0.031	0.143	–

* *p* < 0.05, ** *p* < 0.01. ADHD: Attention Deficit Hyperactivity Disorder; CGI-S: Clinical Global Impression Scale—Severity; Hb: Hemoglobin; T-DSM-IV-S: Turgay’s DSM-IV Based Attention Deficit Hyperactivity Disorder and Disruptive Behavior Disorder Screening Scale; WFIRS-P: Weiss Functional Impairment Rating Scale—Parent Form. r^2^ = explained variance.

**Table 5:** The relationship between ferritin levels and WFIRS-P scores in the ADHD group (*n* = 44).

ADHD WFIRS-P Subscale	R (Ferritin)	r^2^ (Explained Variance)
Ferritin	1.000	.
Family	−0.098	0.010
School	−0.002	0.000
Life Skills	−0.382 *	0.146
Self-Concept	0.014	0.000
Social Activities	−0.024	0.001
Risky Activities	−0.156	0.024

* *p* < 0.05. ADHD: Attention Deficit Hyperactivity Disorder; WFIRS-P: Weiss Functional Impairment Rating Scale—Parent Form.

**Table 6:** The partial correlations between ferritin levels and WFIRS-P subscales in the ADHD group (controlling for T-DSM-IV-S total score) (*n* = 44).

WFIRS-P Subscale	Partial r	r^2^ (Explained Variance)
Family	0.180	0.032
School	0.218	0.048
Life Skills	−0.214	0.046
Self-Concept	−0.031	0.001
Social Activities	0.063	0.004
Risky Activities	0.176	0.031
WFIRS-P Total Score	0.142	0.020

ADHD: Attention Deficit Hyperactivity Disorder; WFIRS-P: Weiss Functional Impairment Rating Scale—Parent Form; T-DSM-IV-S: Turgay’s DSM-IV Based Attention Deficit Hyperactivity Disorder and Disruptive Behavior Disorder Screening Scale. Partial correlations were calculated by controlling for the T-DSM-IV-S total score.

## Data Availability

The data supporting the reported results in this study are available upon reasonable request from the corresponding author. Due to privacy and ethical restrictions, the data cannot be publicly shared.

## Supplementary Materials

The following supporting information can be downloaded at: https://www.mdpi.com/article/10.3390/children12080972/s1, Table S1: Summary of studies examining serum ferritin levels in children with ADHD compared to HC.

## Abbreviations

The following abbreviations are used in this manuscript:

ADHD	Attention Deficit Hyperactivity Disorder
CGI-S	Clinical Global Impression Scale—Severity
DSM-5	Diagnostic and Statistical Manual of Mental Disorders, Fifth Edition
DSM-IV	Diagnostic and Statistical Manual of Mental Disorders, Fourth Edition
HCs	Healthy Controls
Hb	Hemoglobin
K-SADS-PL-DSM-5	Schedule for Affective Disorders and Schizophrenia for School-Age Children—Present and Lifetime Version (DSM-5)
T-DSM-IV-S	Turgay’s DSM-IV-Based Attention Deficit Hyperactivity Disorder and Disruptive Behavior Disorder Screening Scale
WBC	White Blood Cell
WFIRS-P	Weiss Functional Impairment Rating Scale—Parent Form
