# Supplementary material for: Assessing the Impact of Serum Ferritin on Life Skills in Children with ADHD
Source: Children (Basel). 2025 Jul 24;12(8):972. doi: 10.3390/children12080972 (PMC12384314; doi:10.3390/children12080972)
Supplement: Supplementary file 1 [file children-12-00972-s001.zip › children-3703103-supplementary.pdf]

Supplementary Table S1. Summary of studies examining serum ferritin levels in children with ADHD compared to HC

Author (Year)	Sample	Mean or median age in subgroups	Female (%) in subgroup	Ferritin Level in ADHD
Lukovac et al. (2022) [35]	67ADHD,<br>66 HC	10.1 ± 1.4<br>9.9 ± 1.5	0<br>0	↑
Tseng et al. (2018) [16]	Meta-analysis*<br>(17 studies)	-	-	↓
Wang et al. (2017) [11]	Meta-analysis*<br>(11 studies)	-	-	↓
Percinel et al. (2016) [33]	200 ADHD,<br>100 HC	11.0 ± 2.4<br>11.0 ± 3.0	36.5<br>40.0	NS
Donfranceso et al. (2012) [34]	101 ADHD,<br>93 HC	8.9 ± 2.5<br>9.2 ± 3.1	8.9<br>11.8	NS
Present Study	44 ADHD,<br>44 HC	8.56<br>9.00	18.2<br>63.6	↓

ADHD: Attention-Deficit/Hyperactivity Disorder; HC: healthy controls; NS: not significant; ↑: higher ferritin level in ADHD group; ↓: lower ferritin level in ADHD group

* Meta-analyses included pooled results from individual studies.
